# Detecting conserved protein complexes using a dividing-and-matching algorithm and unequally lenient criteria for network comparison

**DOI:** 10.1186/s13015-015-0053-5

**Published:** 2015-06-30

**Authors:** Wei Peng, Jianxin Wang, Fangxiang Wu, Pan Yi

**Affiliations:** The School of Information Science and Engineering, Central South University, Changsha, Hunan People’s Republic of China; The Faculty of Information Engineering and Automation, Kunming University of Science and Technology, Kunming, 650093 Yunnan People’s Republic of China; Division of Biomedical Engineering, Department of Mechanical Engineering, University of Saskatchewan, Saskatoon, SK S7N 5A9 Canada; Department of Computer Science, Georgia State University, Atlanta, GA 30302-4110 USA

**Keywords:** Network alignment, Local network alignment, Conserved protein complexes, PPI networks

## Abstract

The increase of protein–protein interaction (PPI) data of different species makes it possible to identify common subnetworks (conserved protein complexes) across species via local alignment of their PPI networks, which benefits us to study biological evolution. Local alignment algorithms compare PPI network of different species at both protein sequence and network structure levels. For computational and biological reasons, it is hard to find common subnetworks with strict similar topology from two input PPI networks. Consequently some methods introduce less strict criteria for topological similarity. However those methods fail to consider the differences of the two input networks and adopt equally lenient criteria on them. In this work, a new dividing-and-matching-based method, namely UEDAMAlign is proposed to detect conserved protein complexes. This method firstly uses known protein complexes or computational methods to divide one of the two input PPI networks into subnetworks and then maps the proteins in these subnetworks to the other PPI network to get their homologous proteins. After that, UEDAMAlign conducts unequally lenient criteria on the two input networks to find common connected components from the proteins in the subnetworks and their homologous proteins in the other network. We carry out network alignments between *S. cerevisiae* and *D. melanogaster*, *H. sapiens* and *D. melanogaster*, respectively. Comparisons are made between other six existing methods and UEDAMAlign. The experimental results show that UEDAMAlign outperforms other existing methods in recovering conserved protein complexes that both match well with known protein complexes and have similar functions.

## Background

The majority of biological processes are not carried out by a single protein alone but by a group of proteins which physically interact with each other to form protein complexes. It is believed that protein complexes are the building blocks of the cellular machinery and protein–protein interaction (PPI) networks evolve at module level [1]. Consequently, identifying protein complexes of a single species plays a significant role in understanding the underlying mechanism of cellular function, and identifying protein complexes conserved across difference species are helpful for studying biological evolution. Recently, some computational methods have been proposed to identify protein complexes from a single PPI network [2–9]. The underlying hypothesis behind these methods is that a protein complex corresponds to a dense subgraph or cluster of a single PPI network. Meanwhile, some computational methods have been introduced to identify the common subnetworks (conserved functional modules) across species by comparatively analyzing PPI networks of different species.

In contrast to traditional sequence-comparison-based methods, network-comparison-based methods provide a new view of studying biological evolution, which considers two proteins conserved across species if they have both similar sequences and similar interactive patterns. The two proteins (homologous protein pairs) that are from two different PPI networks and have similar sequences are believed to have similar interactive patterns if their neighbors in corresponding PPI networks also have similar sequences. These network-comparison-based methods define the problem as network alignment. In context of biology, there are two challenges exist in PPI network alignment. The one is there exist many-to-many mappings between proteins of different species, which is the result of biological evolution, such as gene duplication [10]. The other is few strict meaning of conserved interactive patterns exist due to emergence or elimination of interactions in the course of evolution.

According to differences in the ways to deal with many-to-many mapping, network alignment can be classified into two categories: global alignment and local alignment [11]. The aim of global alignment is to find one-to-one optimal mappings between proteins of two PPI networks. Global alignment can help us to understand variations between species and be used to detect functions of orthologs and construct phylogenetic relationships. There are also some global alignment methods [12] adopt some clustering methods to detect conserved subnetworks based on the best mappings between the nodes from different PPI networks. However, these methods ignore the facts that there exist duplications of interacting proteins and even whole complexes in a single species. Previous studies observe that a significant fraction of complexes in *S. cerevisiae* (yeast) share strong similarity with each other [13]. By contrast, local alignment is utilized to detect pathways or protein complexes that are conserved across multiple species. There exist many-to-many mappings between proteins of two PPI networks. Note that there are also other global or local alignment methods [14–17] incorporate some biological information, such as functional annotation, protein structure information, protein domain information to find truly homologous proteins and reduce the impacts of many-to-many mappings. This work focuses on local alignment, whose goal is to find conserved complexes across different species only depending on sequence and topological similarity.

Up to now, many local alignment methods have been proposed to detect conserved protein complexes. Generally, there are two types of local alignment methods: alignment-graph-based method and dividing-and-matching-based method. The basic idea of alignment-graph-based method is that false positive protein interactions are rare possible to duplicate in other species and merging two PPI networks being compared according homologous mappings between proteins can filter false positive protein interactions. Alignment-graph-based methods [18–21] usually take two steps to identify conserved complexes. Firstly, a weighted alignment graph is built from two input PPI networks. Each node of the graph is composed of a pair of homologous proteins, one from each network. Each edge of the graph is weighed by certain methods that account for the degree to which an interaction in one PPI network is conserved across species. After that, some clustering methods are adopted to detect conserved protein complexes from the weighted alignment graph. Those existing alignment-graph-based methods differ in the strategies taken to construct alignment graph and to clustering the alignment graph. Dividing-and-matching-based method is an alternatively way of finding conserved complexes, which firstly uses known protein complexes or computational methods to divide one of the two input PPI networks into subnetworks and then maps the proteins in these subnetworks to the other PPI network [22–25]. The motivation underlying this kind of methods is to investigate how those protein complexes that are experimentally or computationally identified from a single species are conserved across species. In recent years, there are available know protein complexes of some species, such as yeast and human, and some computational methods that have good performance of detecting protein complexes from the PPI network of single species [2, 26–30]. All of this make it pressing to design an effective dividing-and-matching-based method to identify conserved protein complexes.

To overcome the challenge that there are few strict meaning of conserved interactions across species, both alignment-graph-based methods and dividing-and-matching-based methods introduce less restrictive definition of conserved interactions in the course of comparison. As for alignment-graph-based method, some methods, such as Network-Blast [18], NetworkPath [31] and Mawish [19], introduce edges in alignment graph if a pair of proteins in one networks is directly connected while their homologous proteins in the other network are indirectly connected. However PHUNKE [20] cancels the requirement of indirect connection between homologous proteins in the other network and connects two nodes in alignment graph if there is at least a pair of proteins in one network is directly connected. AlignNemo [17] adopts less restrict criterion and constructs edges in alignment graph if a least a pair of proteins in one PPI network is directly or indirectly connected. NetAligner [32] adds edges between node pair in alignment graph at a distance greater than 2 and tolerates gaps and mismatch of any length. As for dividing-and-matching-based method, Manikandan et al. [33] have proposed a Match-and-Split algorithm which matches proteins of two networks according to a local matching criterion and splits the whole networks into connected components. This process is recursively implemented on those components and finally outputs conserved complexes. Luqman and Karp [24] have introduced Produles which uses PageRank-Nibble [34] algorithm to partition one of the two input networks and maps these subnetworks to the other network. After that, a local extension is implemented to detect the connected components that consist of the homologous proteins in the other network. According to those connected components, the subnetworks are refined and the connected parts in them are extracted as conserved protein complexes. Obviously, Match-and-Split and Produles algorithm do not match the two networks exactly in their graph structure. However, they only take direct neighbors into account when implementing local alignment, which is so rigid that very few conserved protein complexes are identified. With respect to this, DAMAlign [25] is proposed in our previous work, which takes both dividing-and-matching strategies and the same lenient criteria as AlignNemo to locally extend a pair of homologous protein pairs. That is, in the course of finding common connected components, DAMAlign recruits a pair of homologous proteins if there is at least one path of length not larger than 2 to connect one of node in the homologous protein pair in its corresponding network. The comparisons made by previous studies show that AlignNemo, AlignMCL [21] and DAMAlign succeed in detecting more conserved complexes than previous methods [18, 19], such as Mawish, NetworkBlast,PHUNKEE and Produles. The reason may be considering indirectly connected node pairs in one network is robust against missing interactions in original network. Although NetAligner employs more lax criteria to introduce conserved interactions, it also yields a lot of false positive conserved interactions, which reduces its performance of detecting conserved complexes.

In spite of that previous researchers have done great efforts to improve the performance of their methods by introducing less strict criteria to find conserved interactions, few of them consider the difference of the two input networks and adopt equally lenient criteria on them. In fact, there exist differences between PPI networks of different species in their structures and topologies. The distance between proteins that have homologous proteins in the other PPI network may vary with species. Therefore, in this work, we propose a new dividing-and-matching method named by UEDAMAlign to detect conserved protein complexes via local network alignment. UEDAMAlign, similar to previous dividing-and-matching methods, such as Produles and DAMAlign, partitions one of PPI network into subnetworks and then maps these subnetworks to the other PPI network to find common connected components. In contrast to previous dividing-and-matching methods, UEDAMAlign implements unequal criteria on the two networks to find common connected components with respect to the structural and topological differences of the two networks. That is UEDAMAlign locally extends a pair of homologous proteins if there is a path of length not larger than *l* to connect the homologous protein in the PPI network one or a path of length not larger than *r* to connect the homologous protein in the other network. To evaluate the effectiveness of UEDAMAlign, We carry out network alignment between *S. cerevisiae* and *D. melanogaster*, *H. sapiens* and *D. melanogaster*, respectively. Comparisons are made between other six existing methods and UEDAMAlign whose parameters *l* and *r* are both set to 2. The experimental results show that When UEDAMAlign takes the same lenient criteria as AlignNemo and DAMAlign do, it is superior to other existing methods because it can detect conserved protein complexes that both match well with known protein complexes and have similar functions. Finally, we discuss the effect of parameters *l* and *r* on the performance of UEDAMAlign.

## Methods

The detection process of UEDAMAlign is broadly divided into four steps. At the beginning, several random walking steps are unequally taken on the two input PPI networks to detect some potential mappings between proteins of the two networks. After that, one of the two PPI networks is divided by known protein complexes or computational methods. Then proteins in those subnetworks are mapped to the other PPI network to find their homologous proteins and the connected components of those homologous proteins are extracted from the other PPI networks by using a heuristic approach. The final step of UEDAMAlign is to filter out the predicted conserved complexes that are highly overlapping with others.

### Exploring potential mappings between proteins of two species

In network alignment, the homologous mappings between the proteins of two different PPI networks can be inferred from their sequence-based similarity. Those proteins with similar sequences are most likely to evolve from a common ancestor and thus have similar functions. Moreover, interactive proteins of a single species tend to share common functions. Therefore, we assume that the protein and its neighbors in a single PPI network should map to a common protein in the other PPI network. Since proteins are most likely to share functions not only with their direct neighbors but also with their indirect neighbors, and even with their level *k* neighbors, some potential mappings between proteins of two species can be inferred from their direct, indirect or level *k* neighbors. Furthermore, the level of neighbors with which a protein tend to share functions varies with species due to the structural and topological difference of their PPI networks. Hence, we should infer potential protein–protein mappings from unequal level of neighbors for different species. In this work, we adopt an unbalanced Bi-random walk algorithm to find potential mapping between proteins of two species. This method has also been used in our previous study [35] that gets protein-function associations by walking different number of steps in PPI network and functional interrelationship network. To formally define our method, some variables are introduced in advance.

Let *P*(N*N) and *H*(M*M) be adjacent matrixes of two input PPI networks respectively. *P*(N*N) is row-normalized and *H*(M*M) is column-normalized. The element *p*(*i*, *j*) of matrix *P*(N*N) and *h*(*i*, *j*) of matrix *H*(M*M) is defined as follows.

1 $$\begin{aligned} p(i,j) = \left\{ \begin{array}{ll} {=}\frac{1}{degree(i)}&\quad {if}\ degree(i) > 0 \\ 0 &\quad {otherwise}\end{array} \right\}. \end{aligned}$$

2 $$\begin{aligned} h(i,j) = \left\{ \begin{array}{ll} {=} \, \frac{1}{degree(j)}&\quad {if}\ degree(j) > 0 \\ 0 &\quad {otherwise}\end{array} \right\}. \end{aligned}$$

where degree(*i*) denotes sum of interactions of node *i* .

Let matrix *A*(N*M) represent known protein–protein mappings measured by sequence-based similarities. Its element a(i,j) is 1, if there exists an mapping between protein *i* of one species and protein *j* of the other one, 0 otherwise. *R*(N*M) denotes the final protein–protein mappings. The value of its element r(i,j) represents the probability that protein *i* will be mapped to protein *j*.

Given matrix *P*, *H* and *A*, we want to calculate matrix *R*. Since proteins and their level *k* neighbors in one PPI network may map to the same proteins in the counterpart network, several random walk steps are taken on the two PPI networks, respectively. At each walking step, multiplying *P* on the left and *H* on the right respectively can detect some potential protein–protein mappings (Eqs. 3, 4). Then the weighted average of the multiply results updates matrix *R* (Eq. 5). Consider the difference of the two input networks, the level of neighbors from which the proteins infer mapping information should be different. To address this problem, two parameters (l and r) are adopted to control maximal iteration steps in the two networks. Mathematically, the process can be expressed as Algorithm 1. where t (=1, 2, $\ldots$) represents the walking steps. Matrix *A* storing known protein–protein mappings can regulate the iteration process. The parameter $\alpha$(0$<\alpha <$1) is used to adjust the weight of regulation of network and of prior knowledge stored in Matrix *A* (in this work, $\alpha$ is set to 0.5).  $\lambda _p$ or  $\lambda _h$ are indicators which are 1 if the number of walk steps on PPI network One or Two are less than their thresholds (*l* or *r*), respectively, 0 otherwise. ISORank [11] adopts similar strategy to obtain potential mappings between proteins of two different PPI networks and computes their global network alignment. In ISORank, however, random walks are taken simultaneously on the two networks until the global networks. Actually, ISORank treats the two networks equally. However, Our work separately takes random walks on two networks, which walks only several steps (t is set to 1, 2, $\ldots$) and is convenient for controlling different walking steps taken on the two networks according to their difference in topology and structure. Consequently, our method is more flexible to get protein–protein mappings between two PPI networks.

### Detecting conserved protein complexes from PPI networks

**Figure 1:**
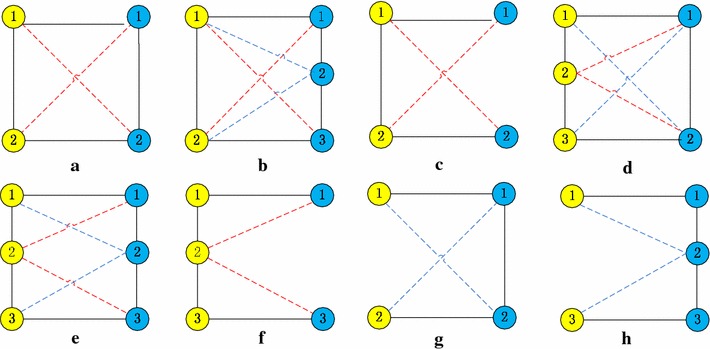
Eight cases (**a**–**h**) of connectivity in conserved protein complexes from two different PPI networks when UEDAMAlign adopts the same lenient criteria as AlignNemo does to extend a pair of homologous proteins. The nodes with *different color* come from different PPI networks. The *full lines* connecting two *different color nodes* represent their known homologous mappings. The *dot lines* represent artificial homologous mappings by a unbalanced Bi-random walk algorithm. The *full lines* connecting the *same color nodes* represent their interactions.

**Figure 2:**
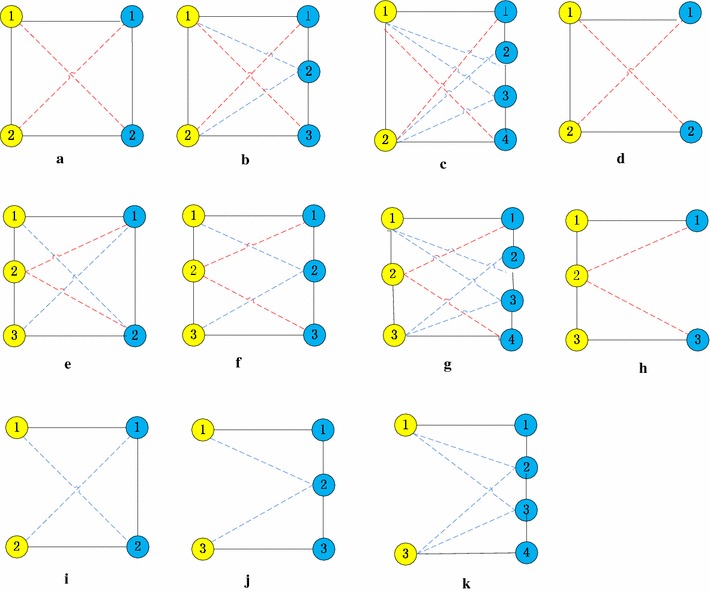
Eleven cases (**a**–**k**) of connectivity in conserved protein complexes from two different PPI networks, when parameters *l* and *r* are set to 2 and 3 in the course of extending a pair of homologous proteins. The nodes with *different color* come from different PPI networks. The *full lines* connecting two *different color nodes* represent their known homologous mappings. The *dot lines* represent artificial homologous mappings by a unbalanced Bi-random walk algorithm. The *full lines* connecting the *same color nodes* represent their interactions.

The basic idea of UEDAMAlign is first dividing PPI networks into small subnetworks and then mapping proteins of subnetworks to the other PPI network. Many computational methods, such as Coach [36], MCL [37, 38], CMC [39], CFinder [40] and so on, have been proposed to detect protein complexes form a single PPI network and achieve good performance. Moreover, biological experiments have been implemented on several species and the data of known protein complexes is available. Consequently those known protein complexes or those predicted by computational methods can be conveniently used as partition of a PPI network. The main challenge of UEDAMAlign lies in mapping proteins in subnetworks of a PPI network to the other one in order to find common connected components. In the course of finding common connected components, UEDAMAlign adopts unequally lenient criteria to extend a pair of homologous proteins. The span distance of a protein pair in a single network is unequal with respect to the difference of input PPI networks, which is determined by inputting parameters *l* and *r*. For example, when taking the same lenient criteria as AlignNemo and DAMAlign do, UEDAMAlign absorbs a pair of homologous proteins into its predicted conserved protein complexes if at least one of protein in the homologous protein pair connects to the proteins in the predicted conserved protein complexes through a path of length not larger than 2. In this case, parameters *l* and *r* are set to 2. When parameters *l* and *r* are set to 2 and 3 respectively, UEDAMAlign locally extends a pair of homologous proteins if there exists a path of length not larger than 2 to connect the node in the homologous protein pair in PPI network one or a path of length not larger than 3 to connect the node in the homologous protein pair in PPI network two. Figure 1 shows eight cases of connectivity in conserved protein complexes from two different PPI networks when *l* and *r* are set to 2. Figure 2 shows eleven cases of connectivity in conserved protein complexes from two different PPI networks when *l* and *r* are set to 2 and 3 respectively. The nodes with different color come from different PPI networks. The full lines connecting two different color nodes represent their known homologous mappings. The dot lines represent artificial homologous mappings detected by unbalanced Bi-random walk algorithm. The full lines connecting the same color nodes represent their interactions.

Given *k* subnetworks $p_1,p_2,\ldots p_k$ extracted from PPI network *P*(N*N), the other PPI network *H*(M*M), known protein–protein mapping matrix *A*(N*M), parameter *l*, *r* and a constructed mapping matrix *R*(N*M), UEDAMAlign proceeds as follows:

Step 1: In this step, we aim to extract the proteins from an input subnetwork that both have homologs in the other PPI network and are connected through at least one path of length not larger than a threshold. The threshold is set to *l* and *r* for network *P* and *H*, respectively. Given ModuleOne and ModuleTwo store conserved protein complexes induced from PPI network *P* and *H* respectively. Start from an arbitrarily node of subnetwork $p_i (i = 1,2,\ldots k)$, find its homologous proteins in *H*, which are homologous to both the node and its neighbors in the input subnetwork $p_i$ according to the matrix *R*. Put the neighbors into ModuleOne if they satisfy one of following conditions:

1. There exists at least one real homologous mapping between the shared homologous proteins and the nodes or its neighbors
2. There exist two different homologous proteins shared by the node and its neighbors but also the two different homologous proteins are really matched with two proteins other than the node and its neighbors in input subnetwork $p_i$.

Since there are some artificial mappings in matrix R, only those real homologous proteins are put into ModuleTwo. The real homologous mappings are stored in Matrix *A*. Then start again from the neighbors, repeat the process in step 1 until no more nodes in subnetworks $p_i$ can been put into ModuleOne.

Step 2: The aim of step 2 is to refine ModuleTwo by reducing many-to-many homologous mappings. Each node in ModuleTwo is assigned a weight, which is defined as sum of mapping values in matrix *R* between the node and its counterpart in ModuleOne. Connected components from proteins in ModuleTwo are deduced by searching both their direct neighbors and up to level *l* or *r* neighbors (level *l* neighbors for subnetworks from network *P*, level *r* neighbors for subnetworks from *H*). For the components that consist of at least two nodes, their counterparts in ModuleOne are regard as being covered. Exclude components with one node from ModuleTwo if their counterparts in ModuleOne have been covered. Otherwise, keep the one with high weight .

Step 3: In this step, we will handle the case that the node of input subnetwork are isolated but their homologous proteins have connections with protein in ModuleTwo. For example, when the parameters *l* and *r* are set to 2, steps 1 and 2 can cover the case of Figure 1a–f. In step 3, we consider the case of Figure 1g, h. When the parameters *l* and *r* are set to 2 and 3, respectively, steps 1 and 2 can cover the case of Figure 2a–h. In step 3, we consider the case of Figure 2i–k. Check the rest of proteins in subnetworks $p_i$ but not in ModuleOne. Attach them to ModuleOne if their counterparts (true homologous proteins in the other PPI network) satisfy one of following conditions.

1. Exist in ModuleTwo.
2. Connect a node in ModuleTwo through a path of length not more than the threshold (*l* for network *P*, *r* for network *H*). In this case, put these counterparts into ModuleTwo.

Since conserved complexes consist of homologous proteins, discard the proteins in ModuleOne or ModuleTwo that have not homologous protein. When all subnetworks in PPI *P* are considered, reverse the role of PPI network *P* and *H*. Input *z* subnetworks ($h_1,h_2,\ldots h_z$) extracted from PPI network *H*, repeat steps 1 to 3.

Step 4: In this step, highly overlapping conserved protein complexes will be filtered out. There are two reasons may contribute to overlap. The one is input subnetworks are overlapping. The other is the homologous mapping between different PPI networks may generate multiple overlapping conserved protein complexes. Comparing two input PPI networks produces a solution consisting of two conserved protein complexes. One comes from each PPI network. The overlap between a pair of solutions is qualified by the overlapping score of their two protein complexes (B and C) from PPI network One. The overlapping score of B and C is defined as follows.

3 $$\begin{aligned} OS(B,C) = \frac{{|{V_B} \cap {V_C}{|^2}}}{{|{V_B}|*|{V_C}|}} \end{aligned}$$

where $V_B$ and $V_C$ denote the node sets of protein complex B and C, respectively. The solution will be filtered out if there exists another solution that consists of larger complex from PPI One and their overlapping score is larger than a threshold t (in this work t = 0.8). In summary, Algorithm UEDAMAlign outlines the overall framework to detect conserved protein complexes by using our method.

## Results

To investigate the effectiveness of our method, first of all, we evaluate the dividing-and-matching strategy of UEDAMAlign. We compare it with other existing methods such as Mawish [19], Networkblast [18], Match-and-Split [33], Produles [24], AlignNemo [17] and AlignMCL [21]. Mawish and Networkblast are two typical alignment-graph-based methods. AlignNemo and AlignMCL are two new alignment-graph-based methods and possess well performance. Match-and-Split and Produles are two dividing-and-matching-based methods. For fair comparison, the parameters *l* and *r* in UEDAMAlign are set to 2, which means UEDAMAlign adopts the same lenient criteria as AlignNemo does and locally extends a pair of homologous proteins if there exists at least one path of length not larger than 2 to connect one of node in the homologous protein pair in its corresponding network. The parameters “a”, “b”, “c”, “d” and “e” in Produles are set to “2”, “100”, “2”, “0.05”, “50” respectively, as recommended by the authors. The threshold of blast E-value used in all comparing methods is set to 10-9. The parameters of other methods are selected as their default values set by the authors. UEDAMAlign explores known protein complexes or some existing computational methods,such as Coach [36], MCL [37, 38], CMC [39], CFinder [40] to partition the PPI networks. The corresponding results are named by UEDAMAlignKnown, UEDAMAlignCoach, UEDAMAlignMCL, UEDAMAlignCMC, UEDAMAlignCFinder, respectively. Among these computational methods that detect protein complexes in a single PPI network, Coach is a very successful clustering algorithm by considering the core attachment structure of protein complex [2]. MCL is a fast and highly scalable clustering algorithm, which partitions a PPI network into non-overlapping subnetworks by simulating a random walker in it. CMC is a clustering method based on Maximal Cliques. CFinder detects the k-cliques in a PPI network and joins two adjacent k-cliques if they share (k$-$1) common nodes. In this work, the parameter k of CFinder is set to 4. The values of parameter of other methods are selected from those recommended by authors.

In this section, we first introduce the experimental data used in this work. Then the performances of the comparing methods are evaluated by matching with known protein complexes. In addition we show the biological relevance of the conserved protein complexes detected by the comparing methods. After that, UEDAMAlign is compared with AlignNemo based on AlignNemo’s experimental dataset. Finally, we show the property of the UEDAMAlign that can take an unequally lenient criteria when comparing two networks. Moreover, the effect of parameters on the performance of UEDAMAlign will be discussed.

### Experimental data

We carry out alignment among two pairs of PPI network, *S. cerevisiae* (yeast) with *D. melanogaster* (fruit fly) and *H. sapiens* (human) with *D. melanogaster*. The PPI network data of yeast and fruit fly is downloaded from DIP database [41], which is published on Oct. 10, 2010, without self-interactions and repeated interactions. There are total of 5,093 proteins and 22,570 interactions in yeast dataset, and 7,916 proteins and 20,289 interactions in fruit fly dataset. The PPI network data of human is obtained from HIPPIE [42], which includes 13,398 proteins and 86,307 interactions, also excludes self-interactions and repeated interactions. The protein sequence data of yeast, fruit fly and human are all downloaded from NCBI. The homologous protein pairs of the two input networks are inferred according to the sequence-based similarity between proteins from different PPI networks. The sequence-based similarity of two protein *a* and *b* is calculated based on their BlAST E-values as follows.

4 $$\begin{aligned} sim(a,b)=(E(a,b)+E(b,a))/2 \end{aligned}$$

where *E*(*a*,*b*) is the minimum BlAST E-value when aligning *a* against *b*. Here, sequence-base similarities are calculated for protein pairs if their Blast E-values are smaller than $10^{-9}$.

The list of known yeast protein complexes is obtained from literature published in Nucleic Acids Research (CYC2008)  [43], which consists of 408 protein complexes. The list of human protein complexes is obtained from CORUM  [44], which consists of 1613 distinct protein complexes composed by no less than two proteins.

### Matching with known protein complexes

To evaluate the performance of each method, we match the predicted conserved protein complexes with known ones. The better the predicted protein complexes match with the known one, the better the performance of the method has. A predicted conserved protein complex is considered to match with known protein complexes if their overlapping score OS (see Eq. 3) is equal to or larger than a threshold (in this work, threshold = 0.2) [18]. Three statistic measures that are widely used to evaluate a result: Precision Recall and F-measure. Precision measures the percentage of predicted protein complexes that match the known complexes. Recall measures the fraction of known complexes that are matched by the predicted conserved protein complexes. F-measure is the harmonic mean of precision and recall. Formally, they are defined as follows.

5 $$\begin{aligned} Precision = \frac{{TP}}{{\mathrm{{TP}} + \mathrm{{FP}}}} \end{aligned}$$

6 $$\begin{aligned} Recall = \frac{{TP}}{{TP + FN}} \end{aligned}$$

7 $$\begin{aligned} F{\text {-}}measure = 2*\frac {{\rm Pr}ecision*{\rm Re}call} {{\rm Pr}ecision + {\rm Re}call} \end{aligned}$$

where TP (true positive) is the number of predicted conserved protein complexes matched by known protein complexes. FP (false positive) is the number of predicted conserved complexes that fail to match with known protein complexes. FN (false negative) is the number of known protein complexes that are not matched by predicted conserved protein complexes. In addition, coverage rate in introduced to measure how many proteins in the known complexes can be covered by the predicted conserved complexes. Let *m* be the number of known protein complexes $T_{ij}$ is the number of proteins in common between *i*th known protein complex and *j*th predicted conserved protein complex. Coverage rate (CR) is the defined as follows.

8 $$\begin{aligned} CR = \frac{{\sum \nolimits _{i = 1}^m {\mathop {\max ({T_{ij}})}\limits _j } }}{{\sum \nolimits _{i = 1}^m {{|KC_i|}} }} \end{aligned}$$

where $|KC_{i}|$ denotes the number of proteins in the *i*th known complex.

Table 1 shows the basic information of results of different methods based on our experimental dataset. Column “conserved pairs” refers to the number of conserved protein complexes pairs generated from alignment of two different PPI network. Since there exists many-to-many mappings between proteins of different PPI networks, the conserved protein complexes in one network may be repeat and match with different ones in the other network. Additionally, a conserved protein complexes in on network may include some repeat proteins which are mapped to different proteins in the other network. Column “distinct complexes (size ≥2)” refers to the number conserved protein complexes in one PPI network after filtering out repeat proteins in one complex and repeat complexes as well as those that consist of only one protein. For example, AlignMCL yields 933 pairs of conserved protein complexes when comparing yeast PPI network against to fly PPI network. 915 out of 933 conserved protein complexes in yeast PPI network and 927 out of 933 conserved protein complexes in fly PPI network are distinct , each of which includes at least two distinct proteins.

**Table 1:** The basic information of results of different methods

Method	Yeast-fly				
	Conserved pairs	Yeast		Fly	
		Distinct complex (size ≥2)	Avg size	Distinct complex (size ≥2)	Avg size
UEDAMAlignCFinder (k=4)	129	129	7.48	129	10.72
UEDAMAlignCMC	128	128	9.65	128	12.89
UEDAMAlignCoach	725	725	5.84	723	4.32
UEDAMAlignknowncomplex	148	148	3.92	146	5.12
UEDAMAlignMCL	862	862	3.16	861	3.23
AlignMCL	933	915	3.22	927	3.79
Match-and-Split	27	27	4.63	27	6.85
Mawish	41	41	2.34	40	3.55
NetworkBlast	191	179	9.12	191	10.86
Produles	95	46	4.09	46	4.39

Methods	Human-fly				
	Conserved pairs	Human		Fly	
		Distinct complexes (size ≥2)	Avg size	Distinct complexes (size ≥2)	Avg size
UEDAMAlignCFinder (k = 4)	238	238	9.38	235	8.31
UEDAMAlignCMC	404	404	9.39	404	8.69
UEDAMAlignCoach	1,538	1,538	5.96	1,519	5.51
UEDAMAlignknowncomplex	515	515	4.11	510	4.48
UEDAMAlignMCL	1,453	1,453	3.77	1,450	3.2
AlignMCL	1,117	1,094	3.25	1,068	3.31
Match-and-Split	53	53	5.26	53	3.83
Mawish	65	61	2.59	55	2.22
NetworkBlast	164	164	9.01	158	7.6
Produles	187	99	3.77	91	3.41

Table 2 shows the comparison of different methods by matching the predicted conserved protein complexes with known protein complexes. When using known complexes to partition PPI network, our method (UEDAMAlignKnownComplex) detects 148 yeast conserved protein complexes (PC) and 515 human conserved protein complexes (PC), respectively when comparing yeast against fly and comparing human against fly. 145 out of 148 yeast conserved protein complexes match at least a known yeast complex (MPC), and 172 known yeast protein complexes match at least a predicted one (MKC). 508 out of 515 human conserved protein complexes match at least a known human complex (MPC), and 821 known human protein complexes match at least a predicted one (MKC). Moreover, UEDAMAlignKnownComplex detects 45 yeast and 158 human conserved protein complexes which share identical proteins with known yeast and human protein complexes, respectively (PM). The F-measure of UEDAMAlignKnownComplex is about 0.55 in alignment of yeast and fly, and 0.56 in alignment of human and fly, which is the highest among all comparing methods. When using computational methods to partition the PPI network, the performance of our methods varies due to their different performance of detecting protein complexes in a single PPI network. UEDAMAlignCoach possesses the second best performance and its F-measure is 0.34 when aligning yeast with fly, which is 0.11, 0.28, 0.27, 0.29, 0.21 higher than AlignMCL, Match-and-Split, Mawish, NetworkBlast and Produles, respectively. When aligning human with fly, the F-measure of UEDAMAlignCoach achieves 0.28, which is 0.17, 0.25, 0.25, 0.23, 0.24 higher than AlignMCL, Match-and-Split, Mawish, NetworkBlast and Produles, respectively. As for coverage rate (CR), UEDAMAlignKnownComplex and UEDAMAlignCoach also possess the first and the second best coverage rate in the two alignments. Here we don’t compare our methods with AlignNemo because AlignNemo cannot output results on our experimental dataset. AlignMCL takes the same strategy of constructing alignment graph as AlignNemo and are more scalable than AlignNemo, which has the best performance among other existing methods, including Match-and-Split, Mawish, NetworkBlast and Produles, in term of F-measure and coverage rate. Both AlignMCL and UEDAMAlignMCL employ MCL method to partition PPI network. The difference is that the former uses MCL after constructing alignment graph while the latter uses it before aligning with the other PPI network. On the whole, UEDAMAlignMCL is litter advanced than AlignMCL because its F-measure is litter higher than that of AlignMCL in two alignments. The CR value of DAMAlignMCL is higher than that of AlignMCL when comparing human against fly, while is almost the same as that of AlignMCL when comparing yeast against fly.

**Table 2:** Comparison of different methods in terms of how well matching with known proteins

Methods	PC	MPC	MKC	Recall	Precision	F-measure	CR	PM
Yeast-fly								
UEDAMAlignCFinder (k = 4)	129	59	66	0.1471	0.4574	0.2226	0.1891	2
UEDAMAlignCMC	128	58	73	0.1476	0.4531	0.2226	0.2068	0
UEDAMAlignCoach	725	207	129	0.4259	0.2855	0.3419	0.3057	4
UEDAMAlignknowncomplex	148	145	172	0.3806	0.9797	0.5482	0.3432	45
UEDAMAlignMCL	862	159	137	0.3698	0.1845	0.2461	0.2401	9
AlignMCL	915	151	162	0.3804	0.165	0.2302	0.2479	9
Match-and-Split	27	12	20	0.03	0.4444	0.0562	0.0641	2
Mawish	41	16	26	0.0402	0.3902	0.0729	0.0318	1
NetworkBlast	179	9	10	0.0221	0.0503	0.0307	0.0391	0
Produles	46	29	26	0.0706	0.6304	0.1269	0.0573	3
Human-fly								
UEDAMAlignCFinder (k = 4)	238	80	187	0.0531	0.3361	0.0917	0.106	3
UEDAMAlignCMC	404	67	182	0.0447	0.1658	0.0705	0.1585	2
UEDAMAlignCoach	1,538	428	493	0.2765	0.2783	0.2774	0.2983	10
UEDAMAlignknowncomplex	515	508	821	0.3908	0.9864	0.5598	0.4242	158
UEDAMAlignMCL	1,453	171	322	0.117	0.1177	0.1173	0.2008	9
AlignMCL	1,094	144	305	0.0992	0.1316	0.1131	0.1697	7
Match-and-Split	53	23	73	0.0147	0.434	0.0285	0.0558	3
Mawish	61	28	70	0.0178	0.459	0.0343	0.0333	1
NetworkBlast	164	45	107	0.029	0.2744	0.0525	0.0897	0
Produles	99	35	77	0.0223	0.3535	0.0419	0.0461	5

### Biological relevance of conserved protein complex pairs

To further validate our method, we investigate biological relevance between the conserved protein complexes from the two different PPI networks, which is measured by the average of functional similarity among all proteins in them. Functional similarity of two proteins refers to the semantic similarity of their GO annotations [45]. Given two protein $p_1$ and $p_2$, and their GO annotations GO($p_1$) and GO($p_2$), the functional similarity between protein $p_1$ and $p_2$ is defined as follows:

9 $$\begin{aligned} sim(p_1,p_2)=max(Resinksim(go_i, go_j)) \end{aligned}$$

where $go_i\in$ GO($p_1$) and $go_j\in$ GO($p_2$). Resinksim($go_i$, $go_j$) refers to the semantic similarity score of GO pair ($go_i$, $go_j$) measured by Resink method [46]. In this work, we use Resinksim to measure the similarity between GO terms because both AlignNemo [17] and AlignMCL [21] use it. Based on Resink method, a free tool FastSemSim (http://sourceforge.net/projects/fastsemsim/) is adopted to calculate the similarity of two proteins. The GO system consists of three separate categories of annotations, namely Molecular Function (MF), Biological Process (BP) and Cellular Component (CC). In this work, we mainly focus on the biological process (BP).

Table 3 shows the comparison of each method in terms of the functional similarity of conserved protein complex pairs, when comparing yeast against fly and comparing human against fly. Column “avg_yeast” and “avg_fly” refer to the average functional similarity of conserved yeast protein complexes and conserved fly protein complexes respectively when comparing yeast against fly. Column “avg_intra” lists the average functional similarity of conserved protein complex pair, when only considering the functional similarity between proteins from different species. Column “avg_mixed” lists the average functional similarity of conserved protein complex pair, when considering the functional similarity among all proteins, both inter-species and intra-species. Results for two alignments show that UEDAMAlignKnownComplex yields conserved protein complex pairs which are highly functional related, due to the highest avg_mixed values. Our method using computational methods, such as Coach, CMC and CFinder, to partition PPI networks, can also produce conserved protein complex pairs with similar functions, because their avg_mixed values for two alignments are higher than that of AlignMCL and NetworkBlast, comparable to that of Produles and litter lower than that of Match-and-Split and Mawish. As for UEDAMAlignMCL, it has relative lower avg_mixed values. However, its avg_mixed value is higher than that of AlignMCL for the alignment between yeast and fly, and comparable to that of AlignMCL for the alignment between human and fly.

**Table 3:** Comparison in terms of biological relevance between each pair of conserved protein complexes predicted by each method

Methods	Yeast-fly				
	*Conserved pairs*	$Avg\_mixed$	$Avg\_yeast$	$Avg\_fly$	$Avg\_intra$
UEDAMAlignCFinder (k = 4)	129	3.96	5.3766	3.4259	3.7503
UEDAMAlignCMC	128	3.5266	4.9468	2.9469	3.3061
UEDAMAlignCoach	725	3.4729	4.4809	2.5707	3.1565
UEDAMAlignKnownComplex	148	4.5041	7.0767	3.7421	3.9779
UEDAMAlignMCL	862	2.3539	3.2412	1.4475	2.3063
AlignMCL	933	2.2563	2.9469	1.255	2.2319
Match-and-Split	27	4.069	5.7868	3.3512	3.614
Mawish	41	4.4942	5.9584	3.7828	4.2566
NetworkBlast	191	2.2865	2.8698	1.8388	2.198
Produles	95	3.4301	6.3427	2.525	2.8541

Methods	Human-fly				
	*Conserved* *pairs*	$Avg\_mixed$	$Avg\_human$	$Avg\_fly$	$Avg\_intra$
UEDAMAlignCFinder (k = 4)	238	3.7826	4.0948	3.7551	3.6892
UEDAMAlignCMC	404	3.5078	4.0341	3.2929	3.3612
UEDAMAlignCoach	1,538	3.5807	4.0532	3.3599	3.4388
UEDAMAlignKnownComplex	515	4.8490	6.1202	4.4131	4.5720
UEDAMAlignMCL	1,453	2.2718	2.4197	1.905	2.2812
AlignMCL	1,117	2.4166	2.5095	1.7317	2.456
Match-and-Split	53	4.0713	4.484	4.6043	3.7865
Mawish	65	4.424	4.8343	4.9263	4.1549
NetworkBlast	164	3.3956	3.7568	3.3467	3.2386
Produles	187	3.8828	4.3098	4.2651	3.7342

Above results show that although previous methods such as Mawish, Produles and Match-and-Split can yield a small amount of conserved protein complexes that both match well with known protein complexes and are highly functional related, UEDAMAlign is able to detect more high quality conserved protein complexes that are functional related, if taking effective strategy to partition PPI network, i.e. inputting known protein complexes or those predicted by effective computational methods, such as Coach.

### Validation based on experimental data of AlignNemo

Our UEDAMAlign method takes the same lenient criteria as AlignNemo does to align two PPI network. The main difference between the two methods lies in whether or not dividing PPI networks before aligning. However, AlignNemo cannot produce results when using our experimental data. For fair comparison, we compare our method with AlignNemo, as well as AlignMCL based on AlignNemo’s experimental data [17]. Table 4 shows the basic information of their results. The results of two alignment in Tables 5 and  6 show that UEDAMAlignKnownComplex outperforms all comparing methods in term of its F-measure, coverage rate and Avg_mixed value, which suggest it can yield high quality conserved protein complexes not only matching well with known protein complexes but also highly functional related to their counterparts. UEDAMAlignCoach possesses the second best performance among all comparing methods in term of their F-measure and coverage rate. Its Avg_mixed value is comparable to UEDAMAlignCFinder (k = 4), UEDAMAlignCMC. As for UEDAMAlignCFinder and AignNemo, UEDAMAlignCFinder (k = 4) divides PPI network by using CFinder to detect the 4-cliques in a PPI network, and AignNemo detects conserved protein complexes from alignment graph by extracting 4-subgraphs. DAMAlignCFinder (k = 4) has higher F-measure and Avg_mixed value than AignNemo and comparable coverage rate to AlignNemo. As for DAMAlignMCL and AlignMCL, both methods use MCL method to partition network before or after aligning two network. UEDAMAlignMCL has higher F-measure and Avg_mixed value than AignMCL and comparable coverage rate to AlignMCL. All of these facts verify the effectiveness of our methods that take the dividing-and-matching strategy to align two networks.

**Table 4:** The basic information of results of different methods based on AlingNemo’s dataset

Methods	Yeast-fly				
	Conserved pairs	Yeast		Fly	
		Distinct complexes (size ≥2)	Avg size	Distinct complexes (size ≥2)	Avg size
UEDAMAlignCFinder (k = 4)	126	126	8.02	126	17.13
UEDAMAlignCMC	127	127	10.57	127	23.39
UEDAMAlignCoach	1,019	1,019	9.34	1,019	18.6
UEDAMAlignknowncomplex	160	160	4.04	156	8.65
UEDAMAlignMCL	697	697	6.26	696	5.35
AlignNemo	248	243	9.27	246	10.06
AlignMCL	684	523	3.63	630	12.92

Methods	Human-fly				
	Conserved pairs	Human		Fly	
		Distinct complexes (size ≥2)	Avg size	Distinct complexes (size ≥2)	Avg size
UEDAMAlignCFinder (k = 4)	116	116	9.74	114	8.86
UEDAMAlignCMC	288	288	9.86	287	9.24
UEDAMAlignCoach	2,978	2,978	14.82	2,968	13.98
UEDAMAlignknowncomplex	333	333	4	312	3.98
UEDAMAlignMCL	679	679	3.45	677	3.11
AlignNemo	114	114	12.27	114	11.94
AlignMCL	732	732	4.68	729	4.18

### Effect of parameters on performance

The other contribution of UEDAMAlign lies in being capable of taking unequally lenient criteria when comparing two PPI networks. It makes use of two parameters *l* and *r* to control the walking steps taken in the two input PPI networks and therefore determine the distance that a protein pair can span in corresponding network. For example, when aligning the network of yeast and fruit fly, setting parameter *l* and *r* to 2 and 3 respectively means that UEDAMAlign locally extends a pair of homologous proteins if there exists one path of length not larger than 2 to connect the yeast node in the homologous protein pair or one path of length not larger than 3 to connect the fruit fly node in the homologous protein pair. Specially, as *l* and *r* are both set to 2, UEDAMAlign achieves the same performance to DAMAlign on detecting conserved protein complexes. To investigate the effect of unequally lenient strategy on the performance of detecting conserved protein complexes, we vary the two parameters ranging from 2 to 3 and evaluate the prediction accuracy of UEDAMAlign when utilizing known protein complexes or Coach to partition the input PPI networks.

Tables 7 and 8 show that in the two alignments, UEDAMAlign does not always possess the best performance when its parameters *l* and *r* are both set to 2 in terms of F-measures values and Avg_mixed values. For example, as aligning human and fruit fly, UEDAMAlignKnownComplex when the parameters *l* and *r* are set to 3 and 2 outperforms that when the parameters *l* and *r* are both set to 2. It means that taking unequally lenient criteria on the two input networks by setting suitable values to the parameters can improve the performance of UEDAMAlign.

**Table 5:** Comparison of different methods in terms of how well matching with known protein based one AlignNemo’s dataset

Methods	PC	MPC	MKC	Recall	Precision	F-measure	CR	PM
Yeast-fly								
UEDAMAlignCFinder (k = 4)	126	62	66	0.1535	0.4921	0.234	0.1682	2
UEDAMAlignCMC	127	57	74	0.1458	0.4488	0.2201	0.2208	0
UEDAMAlignCoach	1,019	190	134	0.4095	0.1865	0.2562	0.288	0
UEDAMAlignknowncomplex	160	158	184	0.4136	0.9875	0.583	0.3745	47
UEDAMAlignMCL	697	113	115	0.2783	0.1621	0.2049	0.2365	4
AlignNemo	243	77	53	0.1782	0.3169	0.2281	0.1755	0
AlignMCL	523	95	97	0.234	0.1816	0.2045	0.2224	5
Human-fly								
UEDAMAlignCFinder (k = 4)	116	42	67	0.0264	0.3621	0.0493	0.0451	1
UEDAMAlignCMC	288	62	101	0.0394	0.2153	0.0666	0.1251	1
UEDAMAlignCoach	2,978	432	281	0.2449	0.1451	0.1822	0.1945	0
UEDAMAlignknowncomplex	333	326	552	0.235	0.979	0.3791	0.2634	34
UEDAMAlignMCL	679	103	219	0.0688	0.1517	0.0947	0.1459	2
AlignNemo	114	31	48	0.0194	0.2719	0.0363	0.0628	0
AlignMCL	732	97	254	0.0666	0.1325	0.0887	0.2012	2

**Table 6:** Comparison in terms of biological relevance between each pair of conserved protein complexes predicted by each method based one AlignNemo’s dataset

Methods	Yeast-fly				
	*Conserved* *pairs*	$Avg\_mixed$	$Avg\_yeast$	$Avg\_fly$	$Avg\_intra$
UEDAMAlignCFinder (k = 4)	126	2.669	4.9984	1.8629	2.5173
UEDAMAlignCMC	127	2.3109	4.669	1.661	2.223
UEDAMAlignCoach	1,019	2.0566	3.784	1.5193	2.0422
UEDAMAlignKnownComplex	160	2.7962	7.16	1.7741	2.4475
UEDAMAlignMCL	697	1.9411	3.0208	1.3032	1.6191
AlignNemo	248	1.7501	3.5919	0.916	1.3803
AlignMCL	683	1.2522	2.283	1.019	1.4451

Methods	Human-fly				
	*Conserved* *pairs*	$Avg\_mixed$	$Avg\_human$	$Avg\_fly$	$Avg\_intra$
UEDAMAlignCFinder (k = 4)	116	3.7834	4.0505	3.583	3.7361
UEDAMAlignCMC	288	3.7938	4.2582	3.6216	3.6653
UEDAMAlignCoach	2,978	3.6269	3.9705	3.4952	3.5129
UEDAMAlignKnownComplex	333	4.8623	5.9331	4.5124	4.5783
UEDAMAlignMCL	679	2.3677	2.3178	2.1294	2.3791
AlignNemo	114	2.7493	2.9498	2.7695	2.5799
AlignMCL	732	2.2485	2.2044	1.7298	2.2649

As Table 7 shown, no matter which one of the two partition methods UEDAMAlign uses, for the alignment of yeast and fruit fly, its highest F-measure values achieve when setting the parameters *l* and *r* to unequal values. Specially, both UEDAMAlignKnownComplex and UEDAMAlignCoach achieve the highest F-measures as the parameters *l* and *r* are set to 2 and 3. For the alignment of human and fruit fly, UEDAMAlign has the highest F-measure values when setting the parameters *l* and *r* to equal values. Specially, UEDAMAlignKnownComplex has the highest F-measures value as the two parameters are set to 2 and UEDAMAlignCoach achieves the highest F-measures value as setting the two parameter to 3. Through analyzing the structure and topology of the three PPI networks, we find that the yeast PPI network contains 5,093 proteins and 22,570 interactions, whose average path length is about 3.84, the fruit fly PPI network contains 7,916 GO terms and 20,289 edges, whose average path length is about 4.5, while the human PPI network includes 13,398 proteins and 86,307 interactions, whose average path length is about 4.2. It is obvious that the PPI network of fruit fly is sparser than that of yeast and is similar dense to that of human, which may cause the difference in criteria for comparing the two pairs of PPI networks.

Table 8 show that the conserved protein complexes that can well match with known protein complexes are less biological relevant.

**Table 7:** Comparison of performance of UEDAMAlignKnownComplex and UEDAMAlignCoach with respect to various values of parameter *l* and *r* on how well matching with known protein

Methods	PC	MPC	MKC	Recall	Precision	F-measure	CR	PM
Yeast-fly								
$UEDAMAlignCoach\_l=2\_r=2$	725	207	129	0.4259	0.2855	0.3419	0.3057	4
$UEDAMAlignCoach\_l=2\_r=3$	762	214	144	0.4477	0.2808	0.3452	0.3078	4
$UEDAMAlignCoach\_l=3\_r=2$	785	217	142	0.4493	0.2764	0.3423	0.3078	4
$UEDAMAlignCoach\_l=3\_r=3$	785	218	144	0.4523	0.2777	0.3441	0.3078	4
$UEDAMAlignKnowComplex\_l=2\_r=2$	148	145	172	0.3806	0.9797	0.5482	0.3432	45
$UEDAMAlignKnowComplex\_l=2\_r=3$	149	146	173	0.3832	0.9799	0.5509	0.3453	46
$UEDAMAlignKnowComplex\_l=3\_r=2$	148	145	172	0.3806	0.9797	0.5482	0.3432	45
$UEDAMAlignKnowComplex\_l=3\_r=3$	149	146	173	0.3832	0.9799	0.5509	0.3453	46
Human-fly								
$UEDAMAlignCoach\_l=2\_r=2$	1,538	428	493	0.2765	0.2783	0.2774	0.2983	10
$UEDAMAlignCoach\_l=2\_r=3$	1,420	410	474	0.2647	0.2887	0.2762	0.2963	9
$UEDAMAlignCoach\_l=3\_r=2$	1,421	401	469	0.2595	0.2822	0.2704	0.2965	9
$UEDAMAlignCoach\_l=3\_r=3$	1,430	406	473	0.2626	0.2839	0.2728	0.2965	9
$UEDAMAlignKnowComplex\_l=2\_r=2$	515	508	821	0.3908	0.9864	0.5598	0.4242	158
$UEDAMAlignKnowComplex\_l=2\_r=3$	521	514	826	0.3951	0.9866	0.5642	0.4269	158
$UEDAMAlignKnowComplex\_l=3\_r=2$	522	515	827	0.3958	0.9866	0.5650	0.4270	158
$UEDAMAlignKnowComplex\_l=3\_r=3$	524	517	829	0.3974	0.9866	0.5666	0.4274	158

**Table 8:** Comparison in terms of biological relevance between each pair of conserved protein complexes predicted by UEDAMAlignKnownComplex and UEDAMAlignCoach with respect to various values of parameter *l* and *r*

Methods	Yeast-fly				
	*Conserved* *pairs*	$Avg\_mixed$	$Avg\_yeast$	$Avg\_fly$	$Avg\_intra$
$UEDAMAlignCoach\_l=2\_r=2$	725	3.4729	4.4809	2.5707	3.1565
$UEDAMAlignCoach\_l=2\_r=3$	762	3.4142	4.5086	2.6173	3.1450
$UEDAMAlignCoach\_l=3\_r=2$	785	3.4591	4.4847	2.6750	3.2003
$UEDAMAlignCoach\_l=3\_r=3$	785	3.4140	4.4826	2.6646	3.1738
$UEDAMAlignKnowComplex\_l=2\_r=2$	148	4.5041	7.0767	3.7421	3.9779
$UEDAMAlignKnowComplex\_l=2\_r=3$	149	4.3174	7.0879	3.6295	3.8850
$UEDAMAlignKnowComplex\_l=3\_r=2$	148	4.4140	7.0767	3.7057	3.9537
$UEDAMAlignKnowComplex\_l=3\_r=3$	149	4.3174	7.0879	3.6295	3.8850

Methods	Human-fly				
	*Conserved* *pairs*	$Avg\_mixed$	$Avg\_human$	$Avg\_fly$	$Avg\_intra$
$UEDAMAlignCoach\_l=2\_r=2$	1.538	3.5807	4.0532	3.3599	3.4388
$UEDAMAlignCoach\_l=2\_r=3$	1.420	3.6842	4.1654	3.4326	3.5286
$UEDAMAlignCoach\_l=3\_r=2$	1.421	3.6766	4.1517	3.4409	3.5189
$UEDAMAlignCoach\_l=3\_r=3$	1.430	3.6762	4.1506	3.4260	3.5201
$UEDAMAlignKnowComplex\_l=2\_r=2$	515	4.8490	6.1202	4.4131	4.5720
$UEDAMAlignKnowComplex\_l=2\_r=3$	521	4.8482	6.1193	4.4186	4.5767
$UEDAMAlignKnowComplex\_l=3\_r=2$	522	4.8567	6.1345	4.4277	4.5760
$UEDAMAlignKnowComplex\_l=3\_r=3$	524	4.8436	6.1261	4.4142	4.5696

For example, as the parameters *l* and *r* are set to 2 and 3, UEDAMAlignKnownComplex and UEDAMAlignCoach achieve the highest F-measures when aligning yeast and fruit fly. However, the conserved protein complexes detected by them under this condition have lower biological relevance duo to the lowest Avg_mixed values. This may be caused by two reasons. The one may be homologous protein pairs with low functional similarity are introduced to identified conserved protein complexes. The other is the proteins in conserved protein complexes have some similar functions with their homologous proteins, which are not found by biologist.

The results in Tables 7 and  8 verify that UEDAMAlign can taking unequally lenient criteria on the two comparing PPI network by setting parameters *l* and *r*. However, it is still a big challenge for us to choose suitable values for parameters *l* and *r* with respect to the difference between the two input networks.

## Conclusion

The aim of this work is to detect protein complexes conserved across species through locally aligning a pair of PPI networks. Most of previous methods adopt equally lenient criteria on the two comparing networks but fail to consider the differences of the two networks. Considering that PPI network has the property of modularity and increasing number of known protein complex data are available, we propose a new dividing-and-matching-based method named by UEDAMAlign to detect conserved protein complexes. UEDAMAlign detects subnetworks from one of PPI network and maps these subnetworks to the other one. After that, UEDAMAlign takes heuristic strategy to find the common connected components from the subnetworks and their homologous proteins in the other network. In the course of finding common connected components, UEDAMAlign takes lenient criteria which may vary with parameters according to topological feature of input PPI networks. To access the effectiveness of UEDAMAlign. we carry out two alignments, yeast with fruit fly, and human with fruit fly. Comparison are made between other existing methods and UEDAMAlign when taking the same lenient criteria as AlignNemo and DAMAlign to extend locally a pair of homologous proteins (parameters *l* and *r* are set to 2). (1) The experimental results shows that UEDAMAlign is superior to all other methods in recovering conserved protein complexes which can both match known protein complexes well and have similar functions if it takes effective strategies to partition PPI networks, for example using known protein complexes or Coach to partition. (2) UEDAMAlignMCL outperforming AlignMCL and UEDAMAlignCFinder outperforming AlignNemo confirm the effectiveness of dividing-and-matching strategy of our UEDAMAlign method. (3) The experimental results when setting various values for the parameters (*l* and *r*) of UEDAMAlign verify that UEDAMAlign can taking unequally lenient criteria on the two comparing PPI network by setting parameters *l* and *r*. However, it is still a big challenge for us to choose suitable values for parameters *l* and *r* with respect to the difference between the two input networks.

## References

[1] Wuchty S, Oltvai ZN, Barabási A-L (2003). Evolutionary conservation of motif constituents in the yeast protein interaction network. Nat Genet.

[2] Li X, Wu M, Kwoh C-K, Ng S-K (2010). Computational approaches for detecting protein complexes from protein interaction networks: a survey. BMC Genomic.

[3] Li M, Wang J, Chen J, Cai Z (2010). Identifying the overlapping complexes in protein interaction networks. Int J Data Min Bioinform.

[4] Li M, Wu X, Wang J, Pan Y (2012). Towards the identification of protein complexes and functional modules by integrating PPI network and gene expression data. BMC Bioinform.

[5] Tang X, Wang J, Liu B, Li M, Chen G, Pan Y (2011). A comparison of the functional modules identified from time course and static PPI network data. BMC Bioinform.

[6] Peng W, Wang J, Zhao B, Wang L (2014) Identification of protein complexes using weighted pagerank-nibble algorithm and core-attachment structure. IEEE/ACM Trans Comput Biol Bioinform. doi:10.1109/TCBB.2014.2343954 10.1109/TCBB.2014.2343954 26357088

[7] Tang X, Wang J, Li M, He Y, Pan Y (2014). A novel algorithm for detecting protein complexes with the breadth first search. BioMed Res Int.

[8] Li M, Chen W, Wang J, Wu F-X, Pan Y (2014). Identifying dynamic protein complexes based on gene expression profiles and PPI networks. BioMed Res Int.

[9] Wang J, Zhong J, Chen G, Li M, Wu F-X, Pan Y (2014) Clusterviz: a cytoscape app for clustering analysis of biological network. IEEE/ACM Trans Comput Biol Bioinform. doi:10.1109/TCBB.2014.2361348 10.1109/TCBB.2014.2361348 26357321

[10] Sharan R, Ideker T (2006). Modeling cellular machinery through biological network comparison. Nat Biotechnol.

[11] Singh R, Xu J, Berger B (2007). Pairwise global alignment of protein interaction networks by matching neighborhood topology. Res Comput Mol Biol.

[12] Atias N, Sharan R (2013). ipoint: an integer programming based algorithm for inferring protein subnetworks. Mol BioSyst.

[13] Yosef N, Kupiec M, Ruppin E, Sharan R (2009). A complex-centric view of protein network evolution. Nucleic Acids Res.

[14] Nguyen P-V, Srihari S, Leong HW (2013). Identifying conserved protein complexes between species by constructing interolog networks. BMC Bioinform.

[15] Vanunu O, Magger O, Ruppin E, Shlomi T, Sharan R (2010). Associating genes and protein complexes with disease via network propagation. PLoS Comput Biol.

[16] Ali W, Deane CM (2009). Functionally guided alignment of protein interaction networks for module detection. Bioinformatics.

[17] Ciriello G, Mina M, Guzzi PH, Cannataro M, Guerra C (2012). Alignnemo: a local network alignment method to integrate homology and topology. PLoS One.

[18] Sharan R, Ideker T, Kelley B, Shamir R, Karp RM (2005). Identification of protein complexes by comparative analysis of yeast and bacterial protein interaction data. J Comput Biol.

[19] Koyutürk M, Kim Y, Topkara U, Subramaniam S, Szpankowski W, Grama A (2006). Pairwise alignment of protein interaction networks. J Comput Biol.

[20] Cootes AP, Muggleton SH, Sternberg MJ (2007). The identification of similarities between biological networks: application to the metabolome and interactome. J Mol Biol.

[21] Mina M, Guzzi P (2014). Improving the robustness of local network alignment: design and extensive assessment of a markov clustering-based approach. IEEE/ACM Trans Comput Biol Bioinform.

[22] Jancura P, Marchiori E (2010). Dividing protein interaction networks for modular network comparative analysis. Pattern Recogn Lett.

[23] Li Z, Zhang S, Wang Y, Zhang X-S, Chen L (2007). Alignment of molecular networks by integer quadratic programming. Bioinformatics.

[24] Hodgkinson L, Karp RM (2012). Algorithms to detect multiprotein modularity conserved during evolution. IEEE/ACM Trans Comput Biol Bioinform (TCBB).

[25] Peng W, Wang J, Wu F (2013) A dividing-and-matching algorithm to detect conserved protein complexes via local network alignment. In: 2013 IEEE international bioinformatics and biomedicine conference (BIBM) on 18–21 Dec. 2013, pp 78–81 (2013)

[26] Wang J, Li M, Chen J, Pan Y (2011). A fast hierarchical clustering algorithm for functional modules discovery in protein interaction networks. IEEE/ACM Trans Comput Biol Bioinform.

[27] Zhao B, Wang J, Li M, Wu F, Pan Y (2014) Detecting protein complexes based on uncertain graph model. IEEE/ACM Trans Comput Biol Bioinform. doi:10.1109/TCBB.2013.2297915 10.1109/TCBB.2013.2297915 26356017

[28] Wang J, Peng X, Peng W, Wu F-X (2014). Dynamic protein interaction network construction and applications. Proteomics.

[29] Wang J, Peng X, Li M, Pan Y (2013). Construction and application of dynamic protein interaction network based on time course gene expression data. Proteomics.

[30] Wang J, Peng X, Xiao Q, Li M, Pan Y (2013). An effective method for refining predicted protein complexes based on protein activity and the mechanism of protein complex formation. BMC Syst Biol.

[31] Kelley BP, Sharan R, Karp RM, Sittler T, Root DE, Stockwell BR (2003). Conserved pathways within bacteria and yeast as revealed by global protein network alignment. Proc Natl Acad Sci.

[32] Pache RA, Céol A, Aloy P (2012). Netaligner-a network alignment server to compare complexes, pathways and whole interactomes. Nucleic Acids Res.

[33] Narayanan M, Karp RM (2007). Comparing protein interaction networks via a graph match-and-split algorithm. J Comput Biol.

[34] Andersen R, Chung F, Lang K (2007). Using pagerank to locally partition a graph. Internet Math.

[35] Peng W, Wang J, Chen L, Zhong J, Zhang Z, Pan Y (2014). Predicting protein functions by using unbalanced bi-random walk algorithm on protein–protein interaction network and functional interrelationship network. Curr Protein Pept Sci.

[36] Wu M, Li X, Kwoh C-K, Ng S-K (2009). A core-attachment based method to detect protein complexes in PPI networks. BMC Bioinform.

[37] van Dongen SM (2000) Graph clustering by flow simulation. Ph.D. thesis, University of Utrecht, The Netherlands

[38] Enright AJ, Van Dongen S, Ouzounis CA (2002). An efficient algorithm for large-scale detection of protein families. Nucleic Acids Res.

[39] Liu G, Wong L, Chua HN (2009). Complex discovery from weighted PPI networks. Bioinformatics.

[40] Adamcsek B, Palla G, Farkas IJ, Derényi I, Vicsek T (2006). Cfinder: locating cliques and overlapping modules in biological networks. Bioinformatics.

[41] Xenarios I, Salwinski L, Duan XJ, Higney P, Kim S-M, Eisenberg D (2002). Dip, the database of interacting proteins: a research tool for studying cellular networks of protein interactions. Nucleic Acids Res.

[42] Schaefer MH, Fontaine J-F, Vinayagam A, Porras P, Wanker EE, Andrade-Navarro MA (2012). Hippie: integrating protein interaction networks with experiment based quality scores. PLoS One.

[43] Pu S, Wong J, Turner B, Cho E, Wodak SJ (2009). Up-to-date catalogues of yeast protein complexes. Nucleic Acids Res.

[44] Ruepp A, Waegele B, Lechner M, Brauner B, Dunger-Kaltenbach I, Fobo G (2010). Corum: the comprehensive resource of mammalian protein complexes. Nucleic Acids Res.

[45] Guzzi PH, Mina M, Guerra C, Cannataro M (2012). Semantic similarity analysis of protein data: assessment with biological features and issues. Brief Bioinform.

[46] Resnik P (1999). Semantic similarity in a taxonomy: an information-based measure and its application to problems of ambiguity in natural language. J Artif Intell Res.

